# **Metabolomic analysis of human cirrhosis, hepatocellular carcinoma, non-alcoholic fatty liver disease and non-alcoholic steatohepatitis diseases**

**Published:** 2016

**Authors:** Akram Safaei, Afsaneh Arefi Oskouie, Seyed Reza Mohebbi, Mostafa Rezaei-Tavirani, Mohammad Mahboubi, Maryam Peyvandi, Farshad Okhovatian, Mona Zamanian-Azodi

**Affiliations:** 1 *Proteomics Research Center, Shahid Beheshti University of Medical Sciences, Tehran, Iran*; 2 *Faculty of Paramedical Sciences, Department of Basic Sciences, Shahid Beheshti University of Medical Sciences, Tehran, Iran*; 3 *Basic and Molecular Epidemiology of Gastrointestinal Disorders Research Center, Research Institute for Gastroenterology and Liver Diseases, Shahid Beheshti University of Medical Sciences, Tehran, Iran*; 4 *Abadan School of Medical Sciences, Abadan, Iran *; 5 *PhysiotherapyResearch Center, Shahid Beheshti University of Medical Sciences, Tehran, Iran*

**Keywords:** Cirrhosis, HCC, NAFLD, NASH, Metabolomics

## Abstract

Metabolome analysis is used to evaluate the characteristics and interactions of low molecular weight metabolites under a specific set of conditions. In cirrhosis, hepatocellular carcinoma, non-alcoholic fatty liver disease (NAFLD) and non-alcoholic steatotic hepatitis (NASH) the liver does not function thoroughly due to long-term damage. Unfortunately the early detection of cirrhosis, HCC, NAFLD and NASH is a clinical problem and determining a sensitive, specific and predictive novel method based on biomarker discovery is an important task. On the other hand, metabolomics has been reported as a new and powerful technology in biomarker discovery and dynamic field that cause global comprehension of system biology. In this review, it has been collected a heterogeneous set of metabolomics published studies to discovery of biomarkers in researches to introduce diagnostic biomarkers for early detection and the choice of patient-specific therapies.

## Introduction

 Hepatic diseases are considered as the liver damage. The diagnostic confirmation of hepatic disease is based on a histological examination or combined results of clinical and imaging examinations (1). Clinical tests include the presence of enzymes in the blood such as alanine transaminase, aspartate transaminase assessment of serum proteins as like serum albumin and serum globulin, prothrombin time, partial thromboplastin time and platelet count (2). However, the mentioned methods cannot be satisfactorily applied to a clinical diagnosis. Not only long time is needed to achieve clinical results, but also blind spots in imaging studies is that they cannot get sensitive diagnoses (3). The liver biopsy is a standard, and an invasive approach to diagnose liver diseases. The early and non-invasive diagnosis of hepatic diseases is a challenging task for the clinician. Considerable efforts have been made to find sensitive and specific predictive markers (4). New techniques and non-invasive diagnostic methods solve these limitations and can be helpful in the early stage diagnosis and may eliminate the requirement for biopsy in patients. Metabolomics, with other omics technologies help detailed understanding of biochemical viral events inside the cell and relationships with each other in the systems biology approach (5-11). Some Metabolomic studies have been reported in biomarker discovery on various diseases in recent years (12-20). Metabolomics is the study of all metabolites with low molecular weight in quantitative scale in unit time under specific environmental conditions in an organism or biological sample. Peptide, alkaloids, nucleic acids, amino acids, organic acids, carbohydrates, vitamins, and polyphenols have been introduced as small-molecule (<1000 Da) in a cell, tissue or organism (21). Metabolomics is reported as a new powerful technology in biomarker discovery and dynamic field that cause global comprehension of biological systems similar to proteomics, transcriptomics and genomics. It is essential to distinguish between diseased and non-diseased status information (22). Obtained results from metabolomic studies have suggested that metabolomic profiles may have the potential for application in the field of disease diagnosis (23, 24) or identification of disease biomarkers (25). Commonly applied techniques in metabolomic analysis are mass spectrometry (MS)-based techniques, including: gas chromatography/mass spectrometry (GC/MS), liquid chromatography/mass spectrometry (LC/MS), nuclear magnetic resonance (NMR) spectroscopy, and Fourier transform infrared (FT-IR) spectroscopy (26, 27). In this review, a brief expression and interpretation about metabolomics studies and its achievements in biomarker discovery of liver diseases are presented. Then, a number of metabolic studies are described that using diverse biological specimens for various types of hepatic diseases including, cirrhosis, hepatocellular carcinoma (HCC), non-alcoholic fatty liver disease (NAFLD) and non-alcoholic steatotic hepatitis (NASH). 

## Metabolomics applications in liver diseases

Early diagnosis of liver diseases is a problem and an obstacle to achieve the best therapy of liver diseases for the clinician. Therefore, non-invasive and simple tests are needed. Metabolomics has been introduced as a way for finding effective diagnostic markers in earlier detection (4). Herein, some published metabolic studies for several liver diseases, including, cirrhosis, HCC, NAFLD and NASH is discussed.

**Cirrhosis**

Liver cirrhosis is a major cause of global health loss, with more than one million deaths in 2010 (28). In cirrhosis, most patients during early disease don’t show specific symptoms of disease and so they may loss in- time therapy. Because, the changes resulting from bridging fibrosis are compensated by liver for this stage of disease. In addition, patients do not show specific symptoms until they enter the stage of decompensation (29). The most important factors for development of cirrhosis are NASH, hepatitis B and C, as well as alcohol consumption. The early diagnosis of cirrhosis is a problem for the clinician and it has been afforded to present sensitive, specific and predictive novel biomarkers that can identify and detect early stage of disease. Actualizing the discovery of biomarkers, new techniques such as metabolomics help to achieve this goal. Some important metabolites in cirrhosis are tabulated in Table 1. 

Investigations on serum metabolic profiling of hepatic cirrhosis by Su-Wen Qi, et al. analyzed metabolites by Nuclear magnetic resonance (NMR) for compensated cirrhosis patients (n = 30), decompensated cirrhosis patients (n = 30) and healthy controls (n = 30). 

Compared with compensated liver cirrhosis (LC) patients, the decompensated LC patients displayed higher levels of pyruvate, phenylalanine, succinate, lysine, histidine, glutamine, alanine, glutamate, creatine and lower levels of acetone, as well as LDL and VLDL. The serum metabolic profiling of cirrhotic patients compared with healthy controls showed decreased levels of lipids and choline, as well as increased levels of glucose and lactate (3). 

Metabolome analysis of plasma was undertaken using NMR in groups of LC and HCC sera detected plasma metabolic profile. Sera metabolomic analysis of healthy humans versus LC and HCC has been shown higher levels of glutamine, Nacetylglycoproteins,acetate, alpha-ketoglutarate, tyrosine,glycerol, 1-methylhistidine and phenylalanine, as well as lower levels of low-density lipoprotein, isoleucine, valine, pyruvate, acetoacetate, creatine, choline and unsaturated lipids (30). Serum metabolic profiling by gas chromatography/mass spectrometry (GC/MS) (Ruyi Xue and colleagues) in hepatite B virus (HBV) infected non-cirrhosis male subjects (n=20) and HBV infected cirrhosis male patients (n=20) showed that glucose, acetic acid, hexanoic acid, D-glucitol, butanoic acid, D-lactic acid, phosphoric acid, 1-naphthalenamine and sorbitol are potential serum biomarkers for HBV infected cirrhosis diagnosis (31). Another investigation on serum metabolomic profiles of cirrhosis and HCC patients using GC/MS and ultra-performance liquid chromatography (UPLC)/MS-MS showed that bile acids and dicarboxylic acids are elevated in cirrhosis. Important pathways for cirrhosis were bile acid metabolism, acylcarnitine metabolism, dicarboxylic fatty acid metabolism, fibrinogen peptide cleavage cascades, haemoglobin catabolism and dipeptide metabolism (details are presented in table 1) (32).

In one study, UPLC-MS is used to analyze the metabolome profile in patients with liver cirrhotic ascites versus healthy controls. Results showed that 5-hydroxytryptamine (5-HT), 5-hydroxyindoleacetic acid (5-HIAA), valin, alanine, leucine, phenylalanine, tryptophan prolin, serine, glutamin, arginine and histidin were remarkably reduced in patients with liver cirrhotic ascites. Tyrosin, glycochenodeoxycholic acid, taurocholic acid, taurodeoxycholic acid, glycoursodeoxycholic, glycochenodeoxy cholic acid, and the ratio of branched amino acid /aromatic amino acid (BCAA/AAA) were significantly increased in these patients. The lysophosphatidylcholines C16: 1, C18: 0 and C18: 2 were suggested as potential biomarkers in patients with liver cirrhosis (33). 

**Table 1:** Summary of recent metabolomic studies in the field of cirrhosis

Author and year	Biological specimens	Technological Platform used	Decreased or increased metabolites in the patients compared to the control group
Qi S-W (2012)	serum	NMR	cirrhotic patients vs healthy controls: glucose, lactate lipid , choline decompensated cirrhosis vs. compensated : pyruvate, phenylalanine, succinate, lysine, histidine, glutamine, alanine, glutamate, creatine acetone , LDL and VLDL
Xue R(2009)	Serum	GC/MS	Glucose, Butanoic acid, Hexanoic acid, Serine, Valine, Urea, Isoleucine, Proline, Galactose , Acetic acid, sorbitol
Gao H(2009)	Serum	NMR	Low-density lipoproteins Isoleucine Leucine Valine pyruvate Acetoacetate Choline Unsaturated lipid Acetate N-acetylglycoproteins Glutamine ketoglutarate Taurine Glycerol Tyrosine 1-methylhistidine Phenylalanine
Fitian AI(2014)	Serum	GC/MS and UPLC/MS-MS	Azelate Undecanedioate 2-hydroxyglutarate Hexadecanedioate Taurochenodeoxycholate (TCDCA) Taurocholate (TCA) Taurocholenate sulphate Glycohyocholate Glycocholate(GCA) Tauroursodeoxycholate Glycochenodeoxycholate (GCDCA) Taurolithocholate 3-sulphate Phenethylamine 1-stearoylqlycerophosphocholine ,glycerophospholipid classes, the glycerophosphoethanolamines and the glycerophosphocholines
Yang T(2014)	Serum	UPLC-MS	5-hydroxytryptamine (5-HT) and 5-hydroxyindoleacetic acid (5-HIAA) alanine, valin, leucin, phenylalanine, tryptophan prolin, serin, glutamin, histidin, arginine tyrosin,glycochenodeoxycholic acid,taurocholic acid,taurodeoxycholic acid,glycochenodeoxy cholic acid,glycoursodeoxycholic acid
Lin X(2011)	Serum	GC/MS	arachidonic acid, cholesterol, ratio of stearic acid to oleic acid,glutamic acid aspartic acid
Shao Y(2014)	urine	LC–QTRAP MS	CIR and HCC vs. controls*: *MTA -deoxy-5-methylthioadenosine and 6-methyladenosinie CIR vs HCC: xanthine, hydroxyphenyllactic acid, L-3-phenyllactic acid, and 1-ribosyl-N-ω-valerylhistamine
Dai W(2014)	urine	LC-MS	steroid hormone
MartínezGranados B(2011)	Liver tissue	NMR	Phosphocholine, Phosphoethanolamine, Glutamate Glutamine, Aspartate ,ß-glucose, unsaturated fatty acids ,free Choline

Red color: increased metabolite

Blue color: decreased metabolite

Down-regulation of the ratio of stearic acid to oleic acid in liver cancer and cirrhosis patients was reported. It is additionally found that the content of arachidonic acid (AA) is remarkably lower in the plasma of patients with hepatitis, cirrhosis and liver cancer relative to controls. The levels of cholesterol in cirrhosis are obviously lower than controls while are stable in hepatitis and liver cancer. This phenomenon might indicate the metabolic disorder of lipoprotein in cirrhosis cases. Glutamic acid decreases significantly in all three types of liver diseases (cirrhosis, HCC, hepatitis), while aspartic acid increases markedly in cirrhosis and liver cancer (34). 

 In another study, potential biomarkers of liver cirrhosis have been reported such as glycocholic acid, glycochenodeoxycholic acid, taurocholic acid, taurochenodesoxycholic acid, and LPCs (35).

Recently, urinary experiment based on liquid chromatography identified potential metabolite biomarkers with comparing 21 CIR cases versus 33 HCC cases and 26 healthy individuals. HCC versus CIR show a significant increase in cyclic AMP, adenosine and several medium-chain acylcarnitines. While, CIR and HCC patients compared with controls show the elevated MTA -deoxy-5-methylthioadenosine and 6-methyladenosinie. Four metabolites including xanthine, L-3-phenyllactic acid, 1-ribosyl-N-ω-valerylhistamine and hydroxyphenyllactic acid had higher urinary levels in the CIR group relative to HCC group. Several markers have been introduced for distinguishing HCC from cirrhosis. For example , carnitine C4:0 and hydantoin-5-propionic acid have been suggested as a combinational marker to discern HCC from CIR. (36).In same way Dai W, et al. found that epitestosterone and allotetrahydrocortisol have a favorable capacity to distinguish HCC from CIR.

Even though there may be common conditions between HCC and cirrhosis such as decreasing urinary steroid hormone pattern in cirrhotic and early HCC patients that was reported (37). 

A few studies have explained metabolome of the hepatic tissue liver for cirrhosis (38). In the compression of cirrhotic tissue with non-cirrhotic tissue, free choline was found to be lower in cirrhotic tissue relative to non-cirrhotic tissue, whereas the amounts of phosphocholine and phosphoethanolamine were found to increase. There were also significant variations between cirrhotic and non-cirrhotic samples in some amino acids. In cirrhosis, glutamine and aspartame were decreased, whereas the levels of glutamate were increased. Other identified metabolites with statistically significant decreases between cirrhotic and non-cirrhotic samples include glucose and some unsaturated fatty acids. 

**Table 2:** Summary of recent metabolomic studies in the field of HCC

Author and year	Biological specimens	Technological Platform used	Decreased or increased metabolites in the patients compared to the control group
Gao H (2009)	serum	NMR	Acetate, pyruvate, glutamine, glycerol, tyrosine, phenylalanine alpha-ketoglutarate, 1-methylhistidine LDL, VLDL, valine, acetoacetate, choline, taurine, "unsaturated lipid"
Fitian AI (2014)	serum	GC/MS UPLC/MS-MS	HCC vs. cirrhosis: 12-hydroxyeicosatetraenoic acid (12-HETE), 15-HETE, sphingosine, γ-glutamyl oxidative stress-associated metabolites, xanthine, amino acids, serine, glycine , aspartate , a-cylcarnitines HCC vs. controls: Azelate, Taurochenodeoxycholate (TCDCA) Taurocholate (TCA), Taurolithocholate 3-sulfate, Grycocholate (GCA) Tauroursodeoxycholate (TDCA) Grycochenodeoxycholate (GDCA) Undecanedioate Sebacate (decanedioate)
Yang Y(2007)	liver	NMR	High-grade HCC vs. low-grade HCC tumors: lactate, leucine glutamine, glutamate, glycine and alanine, choline and phosphorylethanolamine (PE) glucose,PC, GPC, triglycerides and glycogen
Yin P(2009)	serum	HPLCESITOFMS	TCA, GCA, bilirubin, TCDCA, GCDCA, carnitine, acetylcarnitine Hypoxanthine, phytosphingosine, dihydrosphingosine, LPC(18:2), LPC(18:3), LPC(16:1), LPC(18:0), taurine, 6-methyl-nicotinic acid
Chen T(2011)	serum /urine	UPLCESIQTOFMS and GCTOFMS	**Serum:** carnitine, GCDCA, GCA, cysteine, 2-oxoglutarate, lactate, pyruvate, inosine, erythronate, **Urine:** GCA, dopamine, adenosine, xanthine, phenylalanine, dihydrouracil, hypotaurine, threonine, *N* acetylneuraminic acid Serum: glycerol, glycine, serine, aspartate,citrulline, tryptophan, lysine, glucosamine, phenylalanine, β-alanine, glycerate, arabinose, creatinine, phosphate, O-Phospho-l-serine Urine: normetanephrine, Cysteine, TMAO, adenine, cysteic acid, 6-aminohexanoate, creatine
Patterson AD(2011)	plasma	UPLCESIQTOFMS	glycodeoxycholate, deoxycholate 3-sulfate, bilirubin,fetal bile acids 7α-hydroxy-3-oxochol-4-en-24-oic acid and 3-oxochol-4,6-dien-24-oil LPC(20:4), LPC(22:6)LPC(14:0), LPC(16:0), LPC(20:2), LPC(18:0) LPC(18:1), LPC(18:2), LPC(20:5), LPC(18:3), LPC(20:3)
Wang B(2012)	serum	UPLCESIQTOFMS	GCDCA , Canavaninosuccinate, phenylalanine, PC(16:0/22:6, LPC(16:0), LPC(18:0), PC(18:0/18:2), PC(16:0)/20:4)
Ressom HW (2012)	serum	UPLCESIQTOFMS	lysophosphatidylcholine (lysoPC 17:0) glycochenodeoxycholic acid 3-sulfate (3-sulfo-GCDCA), glycocholic acid (GCA), glycodeoxycholic acid (GDCA), taurocholic acid (TCA), and taurochenodeoxycholate (TCDCA)
Xiao JF	serum	UPLCESIQTOFMS	PhePhe TCDCA, GDCA, 3β, 6β-dihydroxy-5β-cholan-24-oic acid, oleyol carnitine
Zhang A	urine	UPLCESIQTOFMS GCA	GCA
Nahon P(2012)	serum	NMR	Glutamate, acetate ,N-acetyl glycoprotein Glutamine
Budhu A(2013)	liver	GCMS	monounsaturated palmitic acid
Beyoğlu D(2013)	liver	GCMS	glucose, glycerol 3- and 2-phosphate, malate, alanine, myo-inositol, and linoleic acid glycolysis, attenuated mitochondrial oxidation, and arachidonic acid synthesis
Chen F(2011)	serum	UPLCESITQMS	1-Methyladenosine
Shariff MI, (2011)	urine	NMR	Creatine, Carnitine Glycine, TMAO, Hippurate, Citrate, Creatinine
Wu H(2009)	urine	GCMS	xylitol and urea elevated.
Yang J(2004)	urine	HPLC	pseudouridine, 1-methyladenosine, xanthosine, 1-methylinosine, 1- and 2-methylguanosine, *N*4-acetylcytidine, adenosine
Chen J(2009)	urine	HILIC RPLC MS	Hypoxanthine, Proline betain, Acetyl carnitine, Carnitine, Phenylacetylglutamine Carnitine C9:1, Carnitine C10:3, Butylcarnitine

Red color: increased metabolite

Blue color: decreased metabolite

## Hepatocellular carcinoma

Hepatocellular carcinoma (HCC) is the third reason of mortality by origin of cancer (39). One of the most primary factors for the poor survival of patients is a late diagnosis of HCC. Thereby, it is important to find sensitive and specific biomarkers for early diagnosis of HCC in medicine (40). 

Metabomic studies provided a suggestion that metabolites are powerful and promising elements for identifying novel biomarkers of HCC (41). It has been reported identification of the potential metabolite biomarkers between high-grade HCC tumors versus low-grade HCC tumors. This research showed increased levels of lactate, leucine ,glutamine, glutamate, glycine, alanine, choline and phosphorylethanolamine (PE) as well as decreased levels of glucose, PC, GPC, triglycerides and glycogen in high-grade HCC (42). Meanwhile, Fitian A and colleagues reported that HCC is related to elevated levels of γ-glutamyl oxidative stress-associated metabolites, hydroxyeicosatetraenoic acids (12-HETE, 15-HETE), sphingosine, xanthine, amino acids serine, glycine and aspartate(32).

Another   study introduced dihydrosphingosine and phytosphingosine as potential and diagnostic biomarkers of HCC with compression of hepatitis Binduced liver cirrhosis and hepatocellular carcinoma's metabolome   (details are shown in table 2) (35). In another study, metabolomic profiles were investigated in sera and urine of HCC (n = 82), benign liver tumor patients (n = 24) and healthy controls (n = 71). The differential metabolites are presented in table 2. Histidine and inosine are two statistical significant metabolites for HCC (43).

 Patterson, et al. found that plasma levels of fetal bile acids, 7α-hydroxy-3-oxochol-4-en-24-oic acid and 3-oxochol-4,6-dien-24-oic acid increase in HCC, and LPSs decrease. Compression of fatty acids quantitative profiles of HCC by two techniques UPLC-ESI-TQMS and GC-MS revealed that in GC-MS HCC- profile, lignoceric acid (24:0) and nervonic acid (24:1) are absent (44). However, the same technique approach on serum profiles from HCC (n = 82), LC (n = 48), and healthy subjects (n = 90), showed that glycochenodeoxycholic acid is an important indicator of HCC diagnosis and disease prognosis (45).

 The finding of comparing urinary metabolic profile of HCC versus the metabolomics profile in early HCC recurrence (one year after operation) introduced that the difference in levels of ethanolamine, lactic acid, acotinic acid, phenylalanine and ribose can introduce for the prediction of early recurrence (46). Ressom, et al. compared sera metabolites of HCC (n=78) and cirrhotic controls (n=184) using ultra performance liquid chromatography coupled with a hybrid quadrupole time-of-flight mass spectrometry (UPLC-QTOF MS). Metabolomic investigation of HCC vs. liver cirrhosis showed that levels of sphingosine-1-phosphate (S-1-P) and lysophosphatidylcholine (lyso PC 17:0) that respectively related on sphingolipid metabolism and phospholipid catabolism increased. Decreasing levels of taurocholic acid (TCA), glycocholic acid (GCA), glycochenodeoxycholic acid 3-sulfate (3-sulfo-GCDCA), glycodeoxycholic acid (GDCA), and taurochenodeoxycholate (TCDCA), which are involved in bile acid biosynthesis (specifically cholesterol metabolism) was reported (47). One MS-based metabolic biomarker discovery study was carried on Egyptian subjects by using ultra performance liquid chromatography coupled with quadrupole time-of-flight mass spectrometer (UPLC-QTOF MS). Compression of metabolic profile of HCC (n=40) versus cirrhosis (n=49) led to identification of decrease of GCA, GDCA, GCDCA, oleyol carnitine, 3beta, 6betadihydroxy- 5beta-cholan-24- oic acid and up regulation of Phe-Phe (48).  In a biochemical study on urine, it has been suggested disruption of primary and secondary steps of bile acid biosynthesis  in HCC patients (49). One NMR based study on sera showed the lipoproteins with higher density were up- regulated in cirrhotic patients without HCC (n=93). Glutamate, acetate, and N-acetyl glycoprotein level in large HCC (n=33) significantly increased. Reported metabolomic profiles of small HCC (n=28) patients were similar to large HCC group. Metabolites that correlated with cirrhosis were lipids and glutamine (50). 

Urinary metabolites from liver cancer patients and healthy volunteers were studied by metabonomic method based on ultra-performance liquid chromatography coupled to mass spectrometry. Both hydrophilic interaction chromatography (HILIC) and reversed-phase liquid chromatography (RPLC) were used to separate the urinary metabolites. Hypoxanthine, Proline betain, Acetyl carnitine, Carnitine and Phenylacetylglutamine were up regulated. However, Carnitine C9:1, and Carnitine C10:3, Butylcarnitine were down regulated (51). Some lipids have been introduced as an agent for progression of hepatocellular carcinoma. In combitional metabolite and gene expression profile study of tumor and non-tumor tissues  showed changes in the expression of stearoyl-CoA-desaturase (SCD) associated with HCC. They introduced SCD as a biomarker for aggressive HCC. In aggressive HCC, levels of monounsaturated palmitic acid, the product of SCD activity, were increased and glucose, glycerol 2- and 3-phosphate, malate, alanine and myo-inositol decreased (52). Beyoğlu D, et al. applied GC-MS to present one panel for biomarker discovery in HCC by comparing tumors and non-tumor liver tissues (the members of each group were 31). HCC was characterized by increasing glycolysis, attenuated mitochondrial oxidation, and arachidonic acid synthesis  versus decreasing of glucose, malate, alanine, glycerol 3-phosphate, glycerol 2-phosphate , myo-inositol, and linoleic acid (53). 1-methyladenosine has been introduced as a serum characteristic metabolite of HCC patients in one study that have been carried on 41 HCC patients versus 38 healthy controls. In this study, inflammatory stress or oxidative DNA damage due to nucleotides modification and hyperactivation of methyltransferases and subsequently increasing of 1-methyladenosine in HCC samples are reported (40). Compression of metabolite profiles of 16, 14 and 17 samples in the HCC, cirrhosis and healthy controlof Egyptian population showed decreasing in glycine, trimethylamine-N-oxide, hippurate, citrate and creatinin as well as increase in creatine and carnitine in HCC patients (54). Evaluation of male urinary metabolome pattern of HCC (n=20) and healthy control (n=20) by GC/MS showed a different amount of expression in Octanedioic acid, Heptanedioic acid, Ethanedioic acid, Glycine, Xylitol, Phosphate, Propanoic acid, Trihydroxypentanoic acid, Primidine, Threonine, Butanedioic acid, Butanoic acid, Hypoxanthine, Tyrosine, Arabinofuranose, Hydroxy proline dipeptide, and Xylonic acid between two mentioned groups (55). Yang J, et al. collected 50 urine samples of healthy control, 27 liver cirrhosis, 30 acute hepatitis, 20 chronic hepatitis and 48 HCC. Compression of obtained metabolite profiles by HPLC has been shown up-regulation of pseudouridine, 1-methyladenosine, xanthosine, 1methylinosine, 1- and 2-methylguanosine and N4- acetylcytidine, adenosine(56). 

**Table 3:** Summary of recent metabolomics studies in the field NAFLD

Reference	Tissue	Platform	Decreased or increased metabolites in the patients compared to the control group
Puri P (2009)	Plasma	HPTLC GCFID LCMS	palmitoleic, oleic acids dihomo gamma-linolenic (20:3n6) acids, Triacylglycerols, palmitoleic acid to palmitic acid (16:0) ratio Linoleic acid
García-Canaveras JC(2011)	liver	UPLCESIQTOFMS	hypoxanthineglutamine, γ-glutamyl-dipeptides creatinine, GCDCA , TCDCA L-glutamyl-L-lysine, Lleucyl-L-proline, glutamate,
Kalhan SC(2011)	plasma	UPLCESITQMS GCMS	carnitine, butyrylcarnitine (C5), glutamyl dipeptides, glutamyl valine, glutamyl leucine, glutamyl phenylalanine and glutamyl tyrosine, methylbutyrylcarnitine, glycocholate, taurocholate, glycochenodeoxycholate, Mannose and lactate glutathione, long-chain fatty acids and cysteine-glutathione
JonathanBarr(2010)	serum	UPLC/MS	lysophosphatidylcholine (LPC),deoxycholic acid bile acids (BAs), sphingomyelin: (SM 36:3), (d18:2/16:0), (d18:2/14:0), (d18:1/18:0), (d18:1/16:0), (d18:1/12:0), and (d18:0/16:0) Creatine

Red color: increased metabolite

Blue color: decreased metabolite

**Table 4:** Summary of recent metabolomic studies in the field NASH

Subject	Tissue	Platform	Decreased or increased metabolites in the patients compared to the control group
Puri P etal(2009)	plasma	HPTLC GCFID LCMS	11-HETE(5-HETE), 8-HETE and 15-HETE docosahexanoic acid (22:6 n3) to docosapentenoic acid (22:5n3) ratio
Kalhan SC(2011)	plasma	UPLCESITQMS GCMS	aspartate, glutamate, phenylalanine, tyrosine, lactate,isoleucine, leucine, valine, acylcarnitines (C3, C4 and C5), γ-glutamyl-tyrosine glucose, xanthine pyruvate, carnitine, butylcarnitin,free carnitin,methylbutylcarnitin, GCA, TCA, GCDCA,glutamyl dipeptide phenylacetate, indolepropionate, cysteine-glutathione disulfide, glycerate, glycerophosphocholine, LPC(18:1), LPC(18:2), LPC(20:4)
Jonathan Barr(2010)	serum	UPLCESIQTOFMS	NASH vs. NAFLD: PC(14:0/20:4), LPC(18:1) NASH vs. NAFLD: LPC(24:0)

Red color: increased metabolite

Blue color: decreased metabolite

## NAFLD/NASH

One type of liver disease is Steatohepatitis (also known as fatty liver disease) that is determined by liver inflammation of with simultaneous fat accumulation in liver (57) . The most prevalent chronic liver disease in western countries is NAFLD. Unfortunately, final diagnosis of NAFLD can only be achieved by liver biopsy and histopathological analysis (58). Different studies have been focused on significant favorable methods to find non-invasive markers of liver disease which can distinguish the early stages of damage (59).  For this purpose, investigators used new techniques such as metabolomics to discover biomarkers that could be useful in diagnosis of NAFLD in early stage (60-63).A small number of individuals with NAFLD willdevelop to more severe stages of liver disease, including NASH (non-alcoholic steatohepatitis). NASH is usually related to the progression of cirrhosis and hepatocellular carcinoma (64).  NASH leads to accumulation of hepatic fat. Chronic inflammation and reactive oxygen species formation occur as a result of disease progression (65, 66). Unfortunately, liver biopsy is the only way to final verification of the NASH diagnosis and grade of development of disease. While, the early diagnosis of NASH in early stage is virtual to prosperous treatment (66). Lipids are important metabolites that play a significant role in liver diseases. According to the analysis of plasma lipids by mass spectrometry, there was a significant increase in total plasma monounsaturated fatty acids driven by palmitoleic (16:1 n7) and oleic (18:1 n9) acids, as well as gamma-linolenic (18:3n6) and dihomo gamma-linolenic (20:3n6) acids in both NAFLD (n = 25) and NASH (n = 50). Levels of palmitoleic acid, oleic acid and palmitoleic acid to palmitic acid (16:0) ratio were significantly increased in NAFLD. While, in NASH subjects   decreasing the docosahexanoic acid (22:6 n3) to docosapentenoic acid (22:5n3) ratio and increasing the level of 11-HETE, a nonenzymatic oxidation product of arachidonic (20:4) acid has been reported (67). Using UPLC-MS, Barr, et al. reported that in NAFLD (n=24) (A parallel animal model /human) level of organic acids, phosphatidylcholine (PC), lysophosphatidylcholine (LPC), Arachidonic acid, Glutamic acid, bile acids (BAs) and sphingomyelin lipids: (SM 36:3), (d18:2/16:0), (d18:2/14:0), (d18:1/18:0), (d18:1/16:0), (d18:1/12:0), and (d18:0/16:0) were changed. The amount of deoxycholic acid was significantly higher in NAFLD versus normal liver (Table 3, 4). Significant changes in serum concentrations of only three phospholipids were reported by compression of NASH serum metabolite with NAFLD (68). In another Metabolomic study by García-Canaveras, et al., 46 samples, 23 from steatotic and 23 from non-steatotic human livers, were analyzed by LC-MS that combines RP and HILIC chromatographic separations. Decreased levels of antioxidant species and higher levels of bile acids and phospholipid degradation products were found in steatotic livers. Also, changes in amino acid metabolism, hypoxanthine, creatinine, glutamate, glutamine, γ-glutamyl-dipeptides concentrations and alterations in energy metabolism were found (69). Plasma Metabolomic analysis by Kalhan SC, et al. showed significant elevation of glycochenodeoxycholate, glycocholate, and taurocholate in NAFLD patients. Concentrations of free carnitine, butyrylcarnitine, and methylbutyrylcarnitine were higher in NASH patients, whereas long-chain fatty acids were lower (details are presented in tables 3 and 4). 

Cysteine-glutathione levels decreased in NASH and steatosis, whereas several glutamyl dipeptides elevated (70).

**Table 5:** Common metabolites that their levels changed among Cirrhosis, HCC, NAFLD and NASH

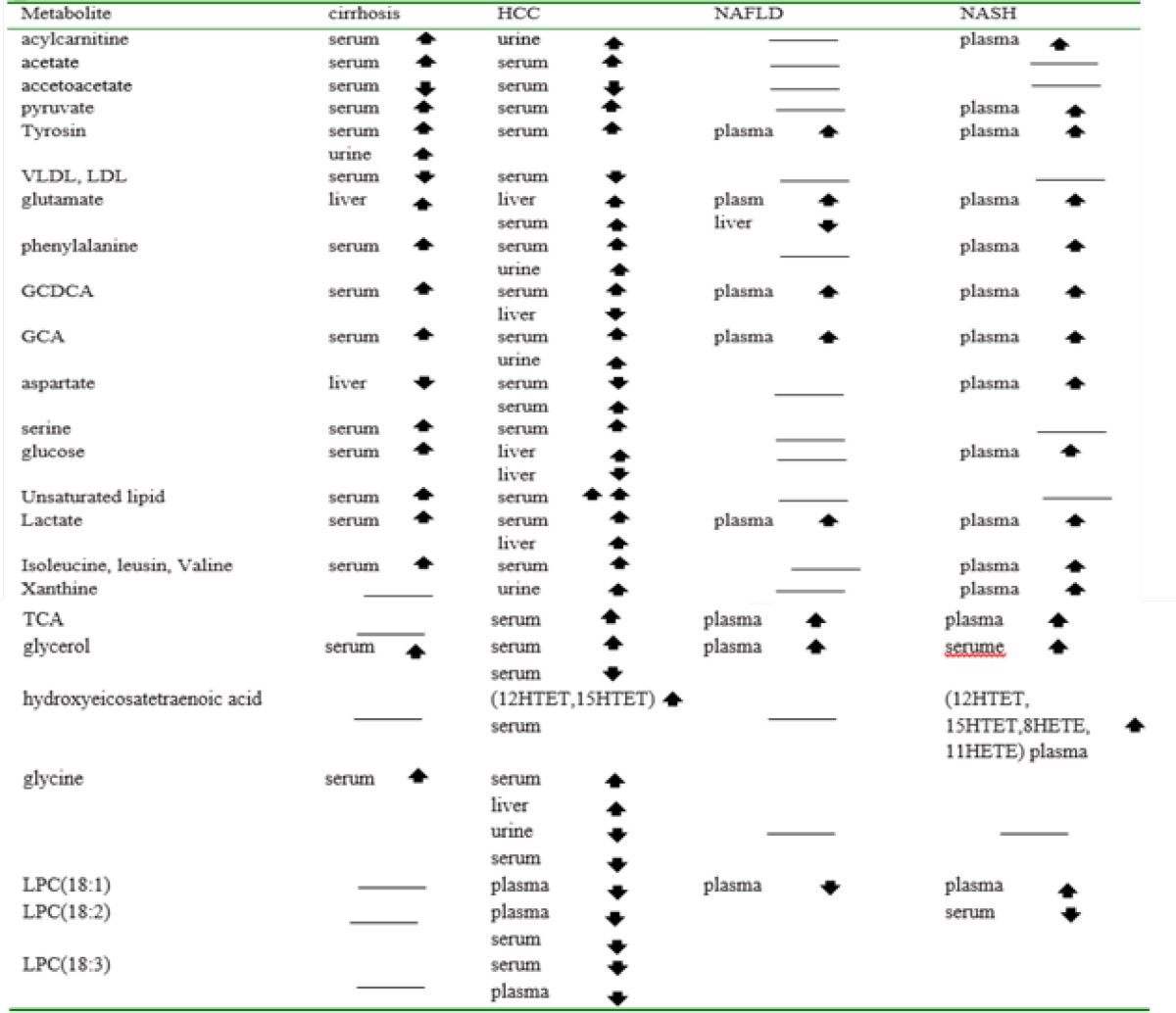

## Major metabolites and metabolic pathways in cirrhosis, HCC, NAFLD and NASH

The common metabolites among cirrhosis, HCC, NASFLD, and NASH diseases are shown in table 5. The reported metabolites are: aromatic amino acids (tyrosine, phenylalanine), hydroxyl amino acids (serine), branched-chain amino acids (valine, isoleucine, leucine), basic amino acids (lysine, arginine), acidic amino acids (glutamate, glycine), aliphatic amino acids (alanine, proline), bile acids, (glycocholate (GCA), glycochenodeoxycholate (GCDCA), taurocholate (TCA) and taurochenodeoxycholate (TCDCA), several carboxylic acids (acetate), ketone bodies (acetoacetate), various choline-associated metabolites (choline, trimethylamine N-oxide (TMAO)), as well as a number of glycolysis and tricarboxylic acid-cycle (TCA cycle) related metabolites (glucose, pyruvate, lactate, succinate, fumarate, and citrate).

The pyruvate concentration elevates in LC and HCC serum. It may be due to reduction of pyruvate utilization into the tricarboxylic acid (TCA) cycle (25). The down –regulated acetoacetate in LC and HCC suggest TCA cycle imperfection and energy metabolism deficiency in liver mitochondria (25). Alteration in concentrations of free amino acids in the LC and HCC serum may occur due to destruction protein followed by cell necrosis. Disordered amino acid metabolism was reported in some metabolomic studies (52,53,71). It has been reported that metabolism of essential amino acids and the common branched-chain amino acids (BCAAs) such as valine, leucine, and isoleucine change in liver diseases (72). The relation of BCAAs with several types of cancer, including HCC has been reported (73). The up-regulated BCAAs in HCC samples may have tumorigenic effect in the liver (74). They have also been connected to other liver diseases such as cirrhosis (73). The fullness of α-ketoglutarate lead to elevated level of glutamine, which afflux of the mitochondria and converted into glutamine in cytosol (30). In cancer cells, the decreased TCA intermediates led by the synthesis of fatty acids and amino acids can be replenished by glutaminolysis (75). Xanthine is produced from hypoxanthine by xanthine oxidase, and the production of xanthine is accompanied by production of H_2_O_2_ (76). The elevated generation of xanthine results in oxidative stress that promotes the development of HCC (26) and NASH (64). 

It is reported that serine elevates in hepatocellular carcinoma (26) and cirrhosis(4) serum. The importance of serine in HCC development may be linked to its secondary function as an allosteric activator of pyruvate kinase M2 (PKM2). PKM2 is the principal cancer isoform of PK that convert phosphoenolpyruvate to pyruvate during glycolysis (77)**. **Glycine is also a fundamental anabolic metabolite of growing cancers and was shown to be elevated in an HCC metabolomics study (55). Lactate is an intrinsic inflammatory mediator. The promotion of chronic inflammation in tumor microenvironments occurs due to   increased interleukin (IL)-17A production by T cells and macrophages that followed by  Lactate (78). However, lactate led to a concentration-dependent increasing the migration of various cancer cell lines (79). Glutamate (a key component in cellular metabolism) participates as a mediator in energy pathways (80). It has been shown the deficiency of glutamate signaling can be effective in the initiation and extension of various cancer types. It was suggested that glutamate is active in stimulation of regulatory pathways that control tumor growth, proliferation and survival(81). High level of acylcarnitine in serum has been confirmed in cirrhosis (26), HCC (26) and NASH (64). In cancer cells, increased glycolysis leads to the elevation of pyruvate level in the cytosol. Pyruvate is transferred into the mitochondria to concede acetylCoA as energy substrate. The increase of acetylCoA leads to increased level of acetylcarnitine (51). Also, due to CPTI catalyzes unification of free fatty acid with carnitine, the efficiency in carbamoyl phosphate transferase I (CPTI) may lead to increased level of acylcarnitines. Aberrant acylcarnitine metabolism has been implicated as an important mechanism of cirrhosis onset (82, 83). It was reported that the strongest difference in metabolite groups between HCC and LC belong to lipid metabolism pathways, amino acid metabolism, eicosanoid signaling, acylcarnitine metabolism, nucleotidemetabolism and oxidative stress homoeostasis (26).

The liver can be considered the hub of lipid metabolism. Numerous HCC metabolomics and genomics analyses have been reported aberrations in lipid metabolism as a signature of HCC development (44, 71, 84, 85). The elevated level of 12-Hydroxyicosatetraenoic acid (12-HETE) and 15-HETE (products of lipoxygenase and cytochrome P450 enzymes) impose both pro and anti-inflammatory effects (86). 12-HETE induce the tumor metastatic effect through activation of protein kinase C (87). 15-HETE is a lipid signaling eicosanoid that acts as endothelial cell adhesion, and promote HCC growth and metastasis through the phosphoinositide 3-kinase/protein kinase B/heat shock protein 90 (PI3K/Akt/HSP90) pathway (88). Members of the 5-HETE family, excite target cells by binding and activating a dedicated G protein-coupled receptor (89). G-protein-coupled receptors (GPCRs), has been introduced as key elements in signal transmission, tumor growth, metastasis (90)  and increased tumor initiation in liver cell (91).

Oxidative stress through accumulation of ROS species superoxide, hydrogen peroxide (H_2_O_2_), and hydroxyl radical induce the development of HCC by amplifying DNA damage (92). H_2_O_2_, a ROS, is released with the oxidation of every dicarboxylic acid (DCA) and during PPAR-α β-oxidation (DCAs are substrates of PPAR-α β-oxidation). DCA and bile acids have been introduced as metabolites that significantly over-expressed in cirrhosis and increase the oxidative stress and free radicals. Also, accumulating levels of free radicals lead to cell death (32). Therefore, oxidative stress induces the apoptosis and inflammatory reactions, which participates in generation of cirrhosis (93). Changes in LPCs regulation were also reported in HCC (35, 53), NAFLD, and NASH (64). LPC not only convert to lysophosphatidic acid by LysoPLD/ATX(LPA) that is involved in cancer development (94), but also is an important signaling molecule involved in regulating cellular proliferation, cancer cell invasion, and inflammation (95). Inflammatory effects include expression alteration of endothelial cell adhesion molecules, growth factors, chemotaxis, and activation of monocytes / macrophages promoted by LPC (96). It has been shown that bile acids (BA) are very useful for cirrhosis diagnosis and hepatobiliary disease (49, 50, 97-99). Dysfunction of bile acid biosynthesis was also reported to be associated with liver cancer progression and development(35, 100). The BA species, including TCA, and CDCA lead to increased levels of cAMP in a dose-dependent manner that influence glucose metabolism(101). However, expression of the genes participate in lipids, liproproteins or glucose metabolism, controlled by bile acids that are physiological ligands for farnesoid X receptors (FXRs) (102). Bile acids are essential molecules in signaling that induce energy consumption by activating intracellular thyroid hormone and regulating energy homeostasis (103). 

## Conclusion

 Finding biomarkers of liver diseases is one of the most serious goals in the modern medicine.  Metabolomics is one of new tools that can apply to earlier disease detection, therapy monitoring and understanding the pathogenesis. In this context, some metabolomics studies were summarized with aim of the identification of metabolites associated with several diseases including cirrhosis, HCC, NAFLD and NASH. In addition, in current review, it has been explained some essential metabolic pathways involved in these diseases, including altered key metabolic pathways such as cellular energy metabolism, bile acids, free fatty acids, glycolysis, and amino acid metabolism, TCA cycle and oxidative stress. There are evidences of deficiency in lipid metabolism, amino acid metabolism, TCA cycle and β-oxidation in liver diseases. Thus, metabolic profiling is an essential step towards the early diagnosis and increasing choice of therapy by introducing special biomarkers involved in the initiation and development of liver diseases. More investigation and biomarker validation is required for application of the finding in medicine.
